# Students’ observations of hand hygiene adherence in 20 nursing home wards, during the COVID-19 pandemic

**DOI:** 10.1186/s12879-022-07143-6

**Published:** 2022-02-14

**Authors:** Ida Hellum Sandbekken, Åsmund Hermansen, Inger Utne, Ellen Karine Grov, Borghild Løyland

**Affiliations:** 1 Department of Nursing and Health Promotion, Oslo Metropolitan University, Postboks 4, St. Olavs Plass, 0130 Oslo, Norway; 2 Department of Social Work, Child Welfare and Social Policy, Oslo Metropolitan University, Oslo, Norway

**Keywords:** Hand Hygiene, Healthcare-associated infections, Nursing homes, Students as observers, Glove use

## Abstract

**Background:**

Healthcare-associated infections are a major threat to patient safety, particularly vulnerable elderly living in nursing homes, who have an increased risk of infections and mortality. Although good hand hygiene is the most effective preventive measure against infections, few studies of hand hygiene adherence have been conducted in nursing homes. The aim of this study is to investigate hand hygiene adherence in nursing homes with students as observers using a validated observation tool. In addition, to examine when healthcare workers perform hand hygiene and when they do not.

**Methods:**

This observational study used the World Health Organization’s observation tool for studying hand hygiene indication and adherence: “My five moments for hand hygiene.” For 1 week each in February and March 2021, 105 first-year nursing students conducted 7316 hand hygiene observations at 20 nursing home wards in one large municipality in Norway.

**Results:**

The overall adherence rate found in this study was 58.3%. Hand hygiene adherence decreased from 65.8% in February to 51.4% in March. The adherence varied largely between the different wards, from 26.4 to 83.1%, and by occupation status, indications of hand hygiene, and use of gloves. Nursing students were found to have the greatest adherence, followed by nurses. The use of gloves reduced adherence. Healthcare workers to a larger degree conduct hand hygiene after contact with patients than before approaching them.

**Conclusions:**

Hand hygiene adherence is too low to protect all residents against healthcare-associated infections, and the findings from this study indicate that there are many factors that influence hand hygiene adherence, eg., education, occupation status and glove use Increasing healthcare workers’ knowledge and skills of hand hygiene is needed to reduce healthcare-associated infections and reminders of the importance of hand hygiene adherence must be an ongoing activity. Interventions to improve hand hygiene adherence in healthcare workers is needed to reduce infections and antibiotic use in nursing homes.

## Background

Healthcare-associated infections (HAIs) are a major threat to patient safety [1] and a major cause of patient morbidity and mortality [2]. HAIs are infections patients acquire when they are receiving care rather than those they had on admission [3]. For the elderly, infection increases mortality, suffering, and hospital stays [4]. Numbers from the Norwegian prevalence survey in November 2020 showed that, at any given time, 3.6% of all nursing home patients had an HAI, varying from 2.9 to 5.3% in different regions [4]. The most effective preventive factor to avoid HAI is hand hygiene, which entails either washing them with soap and water or disinfecting them with alcohol-based hand rubs [5].

The elderly population in Norway is increasing, and the age group of 76–79 years has increased by 47.5% over the last 10 years [6]. With increasing age, the risk of frailty and chronic and multiple diseases rises, which influences physical function, quality of life, and psychological health. The elderly with the most complex health problems often reside in institutions [7]. A review of the literature shows that the elderly have reduced function of their immune systems and are therefore more vulnerable to acute infections [2, 8]. They are also more likely develop infections that need treatment with antibiotics than elderly people living at home [9]. In Norway about 32,000 people live in long-term care nursing homes [10], and are at risk of getting an HAI.

As outlined in a Cochrane review [11], many HAIs can be prevented with good hand hygiene [5]. Nevertheless, studies from nursing homes have shown that hand hygiene adherence varies widely, from 3.6% to 61% [2, 12, 13]. These findings can be explained by differences in healthcare systems between different countries, as well as different healthcare facilities and hospital wards. Results are also influenced by the use of different measurement tools and methods, and it is therefore difficult to compare different studies [14] due to their high heterogeneity [15]. The only study that observed hand hygiene adherence in Norwegian nursing homes using the World Health Organization’s (WHO’s) validated tool for observation [1], found a total adherence of 57% [16]. WHO recommends that the use of observations with a validated observation tool as a method is considered the “gold standard” for assessing hand hygiene adherence [17]. Thus far, few studies have used this method and emphasized hand hygiene adherence in Norwegian nursing homes.

Research on infection control has mainly focused on hospitals and specialist healthcare services [2, 18]. The Norwegian government recently published an action plan with the main goals of reducing the rates of HAI and improving infection control in Norway [19]. This plan includes a focus on hand hygiene, but mainly for hospitals. In nursing homes, surveillance of hand hygiene is only recommended [19], and is justified by the municipality’s right to self-determination. However, the increased number of sick and vulnerable residents with advanced care needs shows that intensifying the focus on infection control is just as important in nursing homes as in hospitals.

In September 2021 in Norway, 814 people had died from the COVID-19 pandemic, and 81% of these were over the age of 70 [20]. In 2020, nearly 57% of deaths occurred in nursing homes [21]. Even though COVID-19 poses a lower threat after vaccination, the statistics show that we need better infection control in nursing homes. The coronavirus is mainly transmitted through droplets from a sick person’s nose or mouth. Droplets can land on surfaces with which people have contact; therefore, frequent, and thorough hand hygiene is one of the most important protective measures against COVID-19 [22]. Focusing on infection control in nursing homes is crucial. Since good hand hygiene is the most effective preventive measure against infections [11], the elderly population is increasing, and the elderly living in nursing homes have reduced immune systems and an increased risk of mortality, a focus on hand hygiene in nursing homes should be a priority.

The aim of this study is to investigate adherence to hand hygiene in nursing homes with the use of observation and a validated observation tool to assess adherence. In addition, this study aims to examine when healthcare workers perform hand hygiene and when they do not.

## Methods

### Design

This study is an observational study of hand hygiene adherence. The study used students in their second semester of a 3-year bachelor’s program in nursing to collect observations. This study was performed according to the STROBE statement. The study is part of a larger research project, which was reviewed by the Regional Committee for Medical and Health Research Ethics, Norway (Ref. 196911 & 226694/REC South-East) and the Norwegian Center for Research Data (Ref. 118936).

### Sample and setting

In December 2020, 17 nursing homes in one municipality in Norway were sent invitation letters to participate in a study on infection prevention from January 2021 to July 2022. Nine nursing homes agreed to participate in a research project involving infection prevention and hand hygiene, which included observations of hand hygiene. A total of 20 wards allowed 105 nursing students from a nearby university to gather observations of hand hygiene in February and March 2021. The quality manager, ward leader, or institution leader signed an agreement for each ward and sent it to the researcher before the study began. Residents had an information sheet delivered to their room and in their mailboxes to their next-of-kin if appropriate. The sheet included information about the study and contact information for the researchers. The healthcare workers received information from their managers. Posters of when the observations would be conducted were posted in the corridors for both residents and healthcare workers to see. In addition, all nursing wards received an educational video of proper hand hygiene, which they were instructed to distribute to all their employees. Because this study is a part of a larger intervention study, an intervention to ensure available alcohol based antiseptic hand rub at all points of care, were not implemented at this stage of the study.

The wards had a mean size of 27 beds (ranging from 18 to 32) and, on average, 22.3 full-time positions (ranging from 13 to 35), with an average of 3.7 full-time nurses’ positions (ranging from 1.7 to 5.3). All staff members in different occupations in each nursing home could be observed. The observations were conducted by two different groups of students at two different periods of time. The first group conducted the observations during week seven in February and the other group during week 11 in March, both in 2021. The weeks were chosen based on when the nursing students had their nursing home placement scheduled. The February group of students was asked to participate as observers after they were placed at a nursing home that took part in the research. The March group was recruited by posting a message on the university’s learning management system, Canvas, and shared it on a private Facebook group the students had access to. The students volunteering to participate were then placed in the nursing wards that took part in the project.

### Education of students conducting the observations

Infection control was a course during all nursing students’ first semester, and they had a 2-h lesson in infection control and hygiene, a 2-h mandatory simulation session in hand hygiene, and an exam in which hand hygiene was a topic. They completed two mandatory courses in infection control, each ending with a test before starting their placement due to the pandemic. All students completed training consisting of 1-h digital lessons about the observations. The students who agreed to participate in this study had an additional 1-h lecture about the observations in Zoom. Both lessons about observations were recorded for availability to watch later. In the lessons, self-made movies of hand hygiene situations were shown in addition to the training videos from WHO [23]. During the lectures, the students were divided into smaller groups in breakout rooms to complete the observation form while watching the movies. The students discussed the content, both in small groups and in plenum, with the researchers.

### Observations

The students conducted the observations during their third of a 4-week practice session in the nursing homes. To reduce the Hawthorne effect, where the observers themselves influence the behavior of those they are observing [24], the students conducted the observations after they were already known to the healthcare workers. Observations were conducted mainly from Monday to Friday. Each student was set up for two observation sessions, each session lasted for 2.5 h, on two different days. They either set up on a morning (8:30–11:00), afternoon (12:00–14:30), or evening session (19:00–21:30). Most students took morning sessions because that was considered the busiest time at the wards. Because of COVID-19 restrictions, most students were not allowed to conduct observations and practice in different wards. Observation session locales were selected on-site according to the health care providers whereabouts.

The students received information that they should contact one of the researchers by e-mail or phone if they had any questions about the observations or the observation form during their week of observation.

The students used the WHO observations form for hand hygiene [1], translated into Norwegian, and used in a prior study [16]. The observation form was based on WHO’s guidelines, “My five moments for hand hygiene,” which defines five key moments when healthcare workers should conduct hand hygiene [17]. The five moments are: 1. before touching a patient, 2. before a clean/aseptic procedure, 3. after body fluid exposure risk, 4. After touching the patient, and 5. after touching patient surroundings. The students reported in a paper form: the occupation of whom they observed, what type of room it was in, which of the five indications for hand hygiene it entailed, hand hygiene action, and whether gloves were used. Data were collected with both a random sampling and a sampling based on knowledge. Occupation and place of observation were collected by random sampling, which means that all occupations and places have an equal probability of being collected. Hand hygiene indication, action and use of gloves were according to WHOs observation form. In accordance with WHOs recommendation, we obtained a total of 21 data points, making our analyzes have a robust estimate [1]. The observation forms were either delivered directly or made anonymous and sent by mail to the first author. From the February group, 56 students conducted observations at 19 nursing wards, and seven students declined to participate. In March, only 17 nursing wards received nursing students, resulting in 49 students conducting observations.

### Analysis

Statistical analyses were performed using IBM® SPSS® for Windows, Version 27. Information from the observation forms was directly coded and plotted into SPSS. Every fourth observation in the datafile was checked for errors. Observations that were wrongfully written or incomplete were not included in the datafile. Descriptive analyses were performed, including crosstabs, Pearson Chi-Square (to assess differences between groups), and McNemar’s test (to assess differences between the two data collection periods). Total adherences were calculated by dividing positive actions with total observed opportunities. Both linear and logistic regressions were conducted with a dichotomous dependent variable to indicate whether the healthcare worker performed hand hygiene or not. Both regression models showed the same significant results, and the results from the linear regression are presented in this article. This decision is based on the arguments made by O Hellevik [25]. Because some independent variables were categorical, dummy variables were used if there were more than two categories.

## Results

### Overall hand hygiene adherence

A total of 7316 indications were observed, and healthcare workers conducted hand hygiene according to recommendations in 4266 of the occasions, for a total adherence of 58.3%. A total of 3513 indications were observed in the February group and 3,803 in the March group. There was a significant (*p* < 0.001) decrease in adherence to hand hygiene between the February group (65.8%) and the March group (51.4%). In 42.2% of the situations, the healthcare workers used hand alcohol based antiseptic, and in 16.1%, they washed their hands. The median for how many indications one student observed was 61, with a range of 13 to 170 indications. In the February group, the median observed indications per student were 52, ranging from 13 to 170, and in March, it was 67, ranging from 15 to 147. From one session the median of observed indications was 30, ranging from 7 to 92. For each ward the median was 359, ranging from 106 to 809. The proportion of adherence was significantly different between the 20 wards (χ^2^ = 277.88, *p* < 0.001, phi = 0.20), with the lowest adherence of 26.4% and the highest of 83.1%. There was no significant difference between the adherence to hand hygiene between the different observation times (morning, afternoon, or evening) (χ2 = 3.40, *p* = 0.183, phi = 0.02).

### Hand hygiene adherence by location, occupation, and indication

As seen in Table 1, there were significant (*p* < 0.001) differences in location, occupation, and indication, as shown in the chi-square tests. The healthcare workers had the highest adherence in the disinfection room (81.7%) and the lowest adherence in the toilet or bathroom (46.2%). The nursing students had the highest adherence (80.5%), and the lowest was found in the unknown occupation group (31.7%). There were differences noted in whether the indication was before contact or after. For all three after-contact indications, adherence was over 65%; for the two before-contact indications, adherence was 46.7% before patient contact and 54.5% before an aseptic task.

**Table 1:** Descriptive table of total observed indications and hand hygiene adherence

	Total observed indications	% (n) preformed hand hygiene
Location		
Disinfection room	619	81.7 (506)
Shared space	778	60.4 (470)
Eating area	1726	58.6 (1011)
Patient room	3585	55.7 (1998)
Toilet or bathroom	608	46.2 (281)
Occupation		
Nursing students	1019	80.5 (820)
Occupational therapists, physical therapists, and bioengineers	80	71.3 (57)
Nurses	2030	67.1 (1362)
Medical doctors	42	59.5 (25)
Nursing assistant	3107	49.5 (1539)
Assistant and High school students	839	47.7 (400)
Unknown	199	31.7 (63)
Indication		
After body fluid exposure risk	958	66.8 (640)
After contact with patient surroundings	1398	65.5 (915)
After patient contact	1848	65.3 (1206)
Before aseptic task	661	54.5 (360)
Before patient contact	2451	46.7 (1145)
Total	7316	58.3% (4266)

Chi-square tests were significant for location, occupation, and indication (*p* ≤ 0.001). *n* number

There was a significant (χ^2^ = 409.97, *p* < 0.001, phi = − 0.237) association between wearing gloves and the use of hand hygiene. Of the workers who used gloves, 64.7% did not conduct hand hygiene according to the WHO recommendations. When not using gloves, only 34.7% did not use hand hygiene as recommended. Gloves were only used in less than 10% of the observations in the dining area, shared space, or disinfection room, in 28% of the observations in the patient room, and in 50% in the toilet or bathroom.

### Regression analyses

Results from the bivariate linear regression analyses supported the findings from the chi-square tests. The only non-significant variables in the regression analysis were status as occupational therapist, physical therapist or bioengineer (*p* = 0.45), or medical doctor (*p* = 0.31; Table 2). In the multivariate model, these results changed when including the interaction terms of place and glove use. In the disinfection room, healthcare workers had a 19% higher probability of conducting hand hygiene than in other rooms. Occupation was significantly associated with hand hygiene adherence. Nursing students had the highest adherence rate, followed by nurses. The “nursing assistants” and “assistants and high school students” groups had a decreased and relatively similar result, while the group of unknown occupations had the lowest adherence.

**Table 2:** A linear probability model using hand hygiene as a dependent variable

Variable	Bivariate analyses			Multivariate analysis		
	B	95% CI	*p*-value	B	95% CI	*p*-value
Constant	–	–	–	0.69	0.63–0.75	< 0.001
Location						
Toilet or bathroom (ref.)						
Patient room	− 0.26	− 0.30 to − 0.22	** < 0.001**	− 0.01	− 0.07–0.04	0.686
Eating area	− 0.23	− 0.28 to − 0.19	** < 0.001**	− 0.01	− 0.06–0.05	0.806
Shared space	− 0.21	− 0.27 to − 0.16	** < 0.001**	− 0.02	− 0.0 to –0.04	0.456
Disinfection room	0.26	− 0.22–0.30	** < 0.001**	0.19	0.13–0.25	** < 0.001**
Occupation						
Nurses (ref.)						
Medical doctors	− 0.08	− 0.22–0.07	0.309	− 0.05	− 0.19–0.09	0.49
Occupational therapists, physical therapists, and bioengineers	0.04	− 0.07–0.15	0.445	0.08	− 0.02–0.18	0.13
Nursing students	0.13	0.10–0.17	** < 0.001**	0.13	0.09–0.16	** < 0.001**
Nursing assistant	− 0.18	− 0.20 to− 0,15	** < 0.001**	− 0.17	− 0.19 to − 0.14	** < 0.001**
Assistant and High school students	− 0.19	− 0.23 to − 0.16	** < 0.001**	− 0.16	− 0.19 to − 0.12	** < 0.001**
Unknown	− 0.35	− 0.42 to − 0.29	** < 0.001**	− 0.34	− 0.41 to − 0.28	** < 0.001**
Indication						
Before patient contact (ref.)						
Before clean procedure	0.08	0.04–0.12	** < 0.001**	0.08	0.04–0.12	** < 0.001**
After contact with patient, surroundings or body fluids	0.12	0.17–0.21	** < 0.001**	0.12	0.01–0.14	** < 0.001**
Time-period						
February (ref.)						
March	− 0.14	− 0.17 to − 0.12	** < 0.001**	− 0.12	− 0.14 to − 0.10	** < 0.001**
Use of gloves						
Not wearing gloves (ref.)						
Wearing gloves	− 0.29	− 0.31 to − 0.26	** < 0.001**	− 0.31	− 0.38 to − 0.24	** < 0.001**
Interaction term: Gloves*Patient room	–	–	–	0.11	0.03–0.19	**0.008**

Significant results are highlighted in bold. Non-significant interaction terms are not showed in the table. *B* the unstandardized beta, *CI* confidence interval

In the first bivariate analyses, there was no significant difference between the three different after-indications, so they were combined into one variable. The indication “before contact with patients” had significantly lower adherence to hand hygiene then “before cleaning procedures” and “after contact with patients, patient surroundings, or contact with body fluids.” The time period (February or March) and the use of gloves were also negative and significant. The probability of correct hand hygiene adherence was reduced by 30.8% with the use of gloves. However, in the patient room, there was an 11% increase in adherence to glove use compared to the toilet and bathroom. The interaction terms for the other locations were not significant. The variables that influenced adherence the most were use of gloves, occupation as a nursing assistant, and whether the observations were conducted in February or March (Standardized β: − 0.25, − 0.17, − 0.12).

## Discussion

To our knowledge, this is the first study to investigate hand hygiene adherence in 20 nursing home wards in Norway.

The overall adherence found in the present study (58.3%) was similar to two other studies. One Norwegian study of nursing homes revealed a 57.2% adherence rate [16], and one French study of different settings for the elderly found an overall adherence rate of 61.5% [12]. It is surprising that the observations conducted during an ongoing pandemic, with an increased focus on infection control and hand hygiene, are similar to observations conducted in 2009 and 2018. All healthcare workers had the opportunity to watch an educational video of how and when to conduct proper hand hygiene, and they knew that they were being observed. Other studies have shown increased hand hygiene adherence when participants know they are being watched, as described by the Hawthorne effect [24].

An adherence of 58% may not be sufficient to prevent all HAIs in nursing homes, so the question is: What is high enough adherence? WHO has suggested that role models in hand hygiene need to have at least 80% adherence [26], but there is little evidence to support this recommendation [15]. Having a too-low acceptable adherence level can cause false safety, but a too-high level can be an unrealistic goal for many and decrease motivation for change. Further research is needed on what an acceptable level of hand hygiene adherence is.

A surprising finding in this study is that the results from the two groups of students showed a significant decrease in hand hygiene adherence, from 65.8% in February to 51.4% in March. There are no other differences between the two groups regarding occupation, glove use, indications, and locations of observations conducted that are of clinal relevance, and these findings are therefore difficult to explain. One explanation may be the COVID-19 pandemic. There was an increasing number of persons infected with COVID-19 in January [20], and there was a pronounced focus on vaccination in nursing homes during January and February [27]. The focus on this disease increases the focus on proper hand hygiene, and may serve as an explanation for the February group’s higher adherence. In March, most residents were vaccinated [28], and the possible pressure to hinder an infection outbreak of COVID-19 may have been lower.

Another possible explanation relates to the different ways in which the two student groups were recruited. The March students may have had higher motivation and knowledge about hand hygiene because they volunteered to participate, whereas the February students were asked to join. The March students may, therefore, have more easily captured the moments where hand hygiene was not conducted according to recommendations.

Another finding that may indicate a greater motivation to conduct observations among the March students is that the March students gathered significantly more (67 indications per student) observations than the February students (52 indications per student). One final explanation may be that hand hygiene adherence is difficult to keep up with over time [29], and increased focus on hand hygiene in January increased adherence in February before it decreased in March.

Hand hygiene adherence has been found to vary significantly between studies, countries, and health facilities [1, 15]. However, evidence also indicates that hand hygiene compliance varies within the same type of healthcare facility and the same region [12]. It is therefore important to assess the representativeness of the included nursing home wards. Conducting observations in 20 nursing home wards, as in this study, should give a representative picture of the nursing homes in that area. Most nursing homes have the same owner, are run in the same way, and all have a quality manager, so it is surprising that hand hygiene adherence varied to the extent found in this study, from 26.4% to 83.1%. These findings indicate that the quality of care can vary greatly between nursing home wards. Occupation may explain some of this variety. In the ward with the lowest adherence, nursing assistants were mostly observed, and in the ward with the highest adherence, nursing students, who were found to have the highest adherence, were mostly observed.

This study found that hand hygiene adherence is largely dependent on the staff’s occupation, and these findings are supported in the literature from nursing homes. One study observing nursing assistants found very low hand hygiene adherence [13], while three other studies comparing nursing assistants with nurses found that nurses had higher adherence than nursing assistants [2, 14, 16, 30]. These findings are supported in this study. Additionally, nurses often have higher adherence than medical doctors [14–16]. This was not significant in the linear regression, but was noted in the chi-square analyses. One reason for this methodological difference may be the small number of observations of medical doctors and occupational therapists, physical therapists, and bioengineers, especially when divided by the other independent variables.

Of our significant results, lower education also resulted in a decrease in adherence. This may indicate that there is an association between the length of education of the staff’s occupation and hand hygiene adherence. Low education may indicate that those with such status are not learning enough about hand hygiene at school or do not have the skills to practice them. To ensure good hand hygiene adherence, a good knowledge base for healthcare workers must be ensured, with a special focus on employees with occupations that have a shorter period of education.

Of the occupations with permanent positions, nurses were found to have the highest adherence. Even so, they were underrepresented as employees in this study. For each nurse position, there were almost three assistants or nursing assistants. An increase in nurse positions may positively influence hand hygiene adherence in nursing homes. On the other hand, it was the nursing students who had the overall highest adherence, which has also been found in another Norwegian study [16]. In a systematic review, nursing students reported greater knowledge and adherence than nurses [31]. Two possible explanations for these findings are the new intensified education in infection control during their education, and the recency of their education. The fact that nursing students have greater adherence than nurses may indicate that infection control is forgotten if the topic is not revisited. This is supported by the literature, which emphasizes the difficulty of creating long-lasting effects of interventions to improve hand hygiene [5, 32, 33]. Staff in nursing homes have often different education and training, and their skills and understanding about the importance of hand hygiene and infection prevention varied greatly [16]. Therefore, reminders of the importance of hand hygiene adherence must be an ongoing activity to all the nursing homes` employees.

One study found an association between location and hand hygiene adherence [16], but they did not conduct a regression analysis. They found that location was only significant for the toilet and bathroom and disinfection room when controlling for the interaction terms of glove use and location. Patient room, dining area, and shared space lost their significance. These results indicate that it is more likely the use of gloves, and not location, affects the probability of hand hygiene adherence.

We found that the probability of using hand hygiene correctly dropped by 30.8% on average when using gloves, and that hand hygiene was not performed in 64.7% of instances. The literature supports the idea that glove use reduces hand hygiene adherence [34–36]. Studies have revealed the misuse of gloves by healthcare workers, in that they wear gloves when they are not recommended, the gloves are not changed as often as they should be [35, 36], two pairs of gloves are worn, or gloves are sanitized [16]. These findings may indicate a lack of knowledge among healthcare workers. To improve hand hygiene adherence, healthcare workers need to increase their knowledge, especially regarding proper glove use.

Previous literature suggests that healthcare workers often use gloves to protect themselves [35] and conduct hand hygiene after contact with patients more often than before [2, 14–16, 37]. The same pattern was found in this study. There was a gap between the “before” and “after” indications, where the three “after” indications had at least 65% hand hygiene adherence, while before patient contact had almost 20% lower (46.7%), and before aseptic tasks had 10% (54.5%) lower adherence. Several studies support these findings, with before-patient contact showing 18.4% to 26.0% lower adherence to hand hygiene than after-patient contact [14, 15, 34]. It seems that healthcare workers perform hand hygiene to protect themselves and not the patients in nursing homes, and that this can be a contributing factor to why nursing home residents get HAIs. These findings indicate that healthcare workers need to improve their knowledge to increase hand hygiene adherence in nursing homes.

## Strengths and limitations

The strengths of this study are the following of WHO recommendations regarding studies on hand hygiene and the use of observation with a validated observation tool. In addition, few studies have conducted more than 7000 observations in nursing homes, with 20 included wards. There are also some potential limitations. First, we found relatively high adherence in this study, which may be explained by the COVID-19 pandemic and increased focus on infection control. However, this is almost the same adherence found in a study from Norwegian nursing homes in 2020 [16]. Second, society was influenced by many COVID-19 restrictions, which meant that none of the researchers were allowed inside the nursing homes, and the distance may have affected students’ observations. Third, the students did not have to pass any tests or exams of knowledge or skills before they conducted the observations. It was based on trust that the students who agreed to take part in the project would contact the researcher if they were unsure of how they should observe. Last, when using an observation method, you need to consider the Hawthorne effect. The ones being observed may have improved their hand hygiene adherence, because someone was watching them [24].

## Conclusion

This study explored hand hygiene adherence in 20 nursing home wards in one municipality in Norway. The overall adherence to hand hygiene was 58.3%, but the findings from this study indicate that there are many factors that influence hand hygiene adherence. Even though the observations were conducted in the same wards in the same municipality in Norway, hand hygiene adherence decreased from 65.8% in February to 51.4% in March, and there were significant differences between different nursing wards, from 26.4% to 83.1%. Findings indicate that occupation and glove use are highly related to hand hygiene adherence. The results from this study also indicate that healthcare workers more often conduct hand hygiene after contact with patients or patient surroundings, which may indicate that they want to protect themselves more than residents. Hand hygiene adherence is too low to protect all residents against HAIs, and a decrease in hand hygiene adherence, despite an ongoing pandemic, shows the need for interventions to improve hand hygiene. Findings from this study indicate that there are many factors that influence hand hygiene adherence, eg. education, occupation status and glove use. Increasing healthcare workers’ knowledge and skills on hand hygiene is needed to reduce healthcare-associated infections and reminders of the importance of hand hygiene adherence must be an ongoing activity. Interventions to improve hand hygiene adherence in healthcare workers is needed to reduce HAI and antibiotic use in nursing homes.

## Data Availability

The data that support the findings of this study are available on request from the corresponding author BL. The data are not publicly available due to them containing information that could compromise research participant privacy.

## Abbreviations

HAI: Healthcare-associated infections
WHO: World Health Organization
NIPH: Norwegian Institute of Public Health
